# High-efficiency retention of ultrafine aerosols by electrospun nanofibers

**DOI:** 10.1038/s41598-022-24739-9

**Published:** 2022-12-02

**Authors:** Yury Salkovskiy, Aleksandr Fadeev

**Affiliations:** Department of Biomechanics, University of Nebraska at Omaha, 6160 University Drive South, Omaha, NE 68182 USA

**Keywords:** Nanoscale materials, Synthesis and processing, Occupational health

## Abstract

The versatility of nanofibrous polymeric materials makes them attractive for developing respiratory protective equipment. Ultrafine nanofibers effectively trap the most penetrating aerosols and exhibit consistent performance compared to conventional electret filters. Advanced nanofiber manufacturing technologies such as electrospinning can functionalize filter materials, enhancing them with unique antibacterial, catalytic, sensory, and other properties. Much of the current research in nanofibrous air filtration focuses on using nanofibers for lightweight personal protective equipment such as N95 respirators, but their use for higher levels of respiratory protection required for chemical, biological, radiological, and nuclear (CBRN) protection has not yet been comprehensively explored. In this study, we tested the hypothesis that electrospun filters could provide the particle filtration efficiency and breathing resistance required by the National Institute for Occupational Safety and Health Standard for CBRN air-purifying respirators. Our manufactured nanofibrous filters demonstrated submicron aerosol retention efficiency of > 99.999999%, which is four orders of magnitude better than the requirements of the CBRN standard. They also had a breathing resistance of ~ 26 mmH_2_O, which is more than twofold lower than the maximum allowable limit. Although the filter material from the gas mask cartridge currently in service with the U.S. military demonstrated a higher quality factor than electrospun filters, the comparative analysis of filter morphology suggested ways of improving nanofibrous filter performance by tuning nanofiber diameter distribution.

## Introduction

Purifying the inhaled air using fibrous filters is the most common and convenient way to protect the respiratory system from harmful airborne chemical particles, smoke, radioactive dust, and pathogens. New emerging threats require the ever-evolving improvement in the effectiveness of personal protective equipment (PPE) through the development of new filter materials with advanced particle retention properties^1^.

Improving PPE performance is associated with a challenging dichotomy between protection and low breathing resistance. The protective properties of filters are evaluated by the filtration efficiency of aerosols with the most penetrating particle size (MPPS), which characterizes the diameter of the aerosol most poorly retained by the filter. The MPPS depends on many parameters of both the filter and the aerosol but typically is 0.02–0.4 µm^2,3^. Interception and diffusion are predominant among the main mechanical mechanisms for capturing MPPS particles by fibrous filters, while the contribution of inertial impact and gravitational sedimentation is negligible due to the small mass of particles. Filtration theory suggests that both interception and diffusion mechanisms increase as the fiber diameter and the resulting inter-fiber spacing decrease^4^. A consequence of the lower inter-fiber spacing is a higher air friction force, resulting in a greater pressure difference (also called pressure drop) needed to maintain the required airflow through the fibrous medium. The higher pressure drop results in additional breathing effort for the wearer of the respirator, which limits the time it can be continuously worn without rest. The National Institute for Occupational Safety and Health (NIOSH) Standard for chemical, biological, radiological, and nuclear (CBRN) full facepiece air-purifying respirators with respirator-mounted replaceable cartridges (canisters) requires inhalation resistance not to exceed 65 mm H_2_O at 85 L/min at the start of use and 80 mm H_2_O at the end of service life^5^.

One of the most promising technologies for creating materials for aerosol filtration is the electrospinning of continuous polymer nanofibers with a diameter of 50–500 nm^6^. Electrospinning is a top-down nanomanufacturing method, where the formation of nanosized structures (one-dimensional continuous nanofibers) is controlled by changing external macroscopic parameters—electric field, air humidity, temperature, feeding rate, etc^7,8^. The global market for electrospun nanofibrous materials has been gradually growing since the invention of needleless electrospinning as a high-performance multi-jet technology for the industrial production of nanofibers^9,10^.

Electrospun filter media are attractive due to their vast possibilities for modifying nanofibers’ structure and chemical composition to give them additional functional properties^11–14^. The use of electrospinning technology for fine air purification is expanding rapidly^15,16^, and recently, there has been increased attention to the electrospinning of nanofibrous filters for personal protective equipment^12,16–20^ due to the COVID-19 pandemic^21,22^. Conventional lightweight PPE, such as the well-known N95 half facepiece masks, cleans the inhaled air from aerosols with electret fibrous materials such as meltblown, and has an MPPS aerosol retention efficiency of 95–99.9%. The diameter of the synthetic polymer fibers in the meltblown material is 2–6 μm^23,24^. These thick fibers cannot effectively capture MPPS aerosols by mechanical filtration mechanisms, so their filtration efficiency primarily depends on the electrostatic charge and drops dramatically when the charge is neutralized due to prolonged improper storage or decontamination required for repeated use^25,26^. Unlike meltblown, electrospun nanofibrous filters are not significantly affected by the loss of electric charge and retain their filtration efficiency after cleaning, making the reusing of light nanofibrous respirators possible without the additional recharging procedures^12,27^.

But despite extensive research in light protective equipment that could replace N95 and similar types of respirators, relatively little attention has been paid to electrospun filters for the PPE with an aerosol retention efficiency of > 99.99%, necessary for a high level of CBRN protection of military and civilian personnel^5,28^. The highest recently reported efficiency of aerosol filtration with electrospun filters was 99.997% under test conditions that did not meet the requirements of the standard for CBRN respirators^29^. In this study, we hypothesized that electrospun filters could provide filtration efficiency of up to 99.999999% while maintaining a pressure drop within the NIOSH-restricted limit for CBRN air-purifying respirators of 65 mm H_2_O at airflow through the filter canister of 85 L/min. To test this hypothesis, we manufactured filters from polymer nanofibers with different nanofiber diameter distributions and area densities using the needleless electrospinning technology and studied their morphology, aerosol permeability, and pressure drops. We used polyacrylonitrile (PAN) for filter manufacturing because it is inexpensive and widely used in electrospinning research^30–32^, which allows results comparison and commercial applications. In addition, we made and tested nanofibrous filters from commercially available polyvinylidene fluoride (PVDF), as this polymer is also widely used to produce electrospin fibers for a variety of applications^7,32,33^. Results were compared to the filter material from a CBRN-grade filter cartridge for Joint Service General Purpose Mask/M50 series protective masks currently in service with the U.S. military^34^.

## Materials and methods

### Materials

Nanofibrous filters were manufactured using polyacrylonitrile yarn (Spinrite, NC, USA) as raw material without additional purification. Anhydrous N,N-dimethylformamide (DMF, cat.# 227056) and acetone (cat.# 179124) were purchased from MilliporeSigma (St. Louis, MO, USA). The polyvinylidene fluoride (Kynar 761) was kindly provided by Arkema Inc. (King of Prussia, PA, USA). Polypropylene spunbond textile (Oxco Inc., SC, USA) with an area density of 40 g/m^2^, typical for producing disposable personal protective equipment^35^, was used as substrate material for nanofibrous filter layers. The comparative filter material was extracted from the Avon CBRNCF50 Protective Mask Filter canister (Avon Protection, MI, USA) used in the Joint Service General Purpose Mask/M50 gas mask.

### Manufacturing

**Figure 1:**
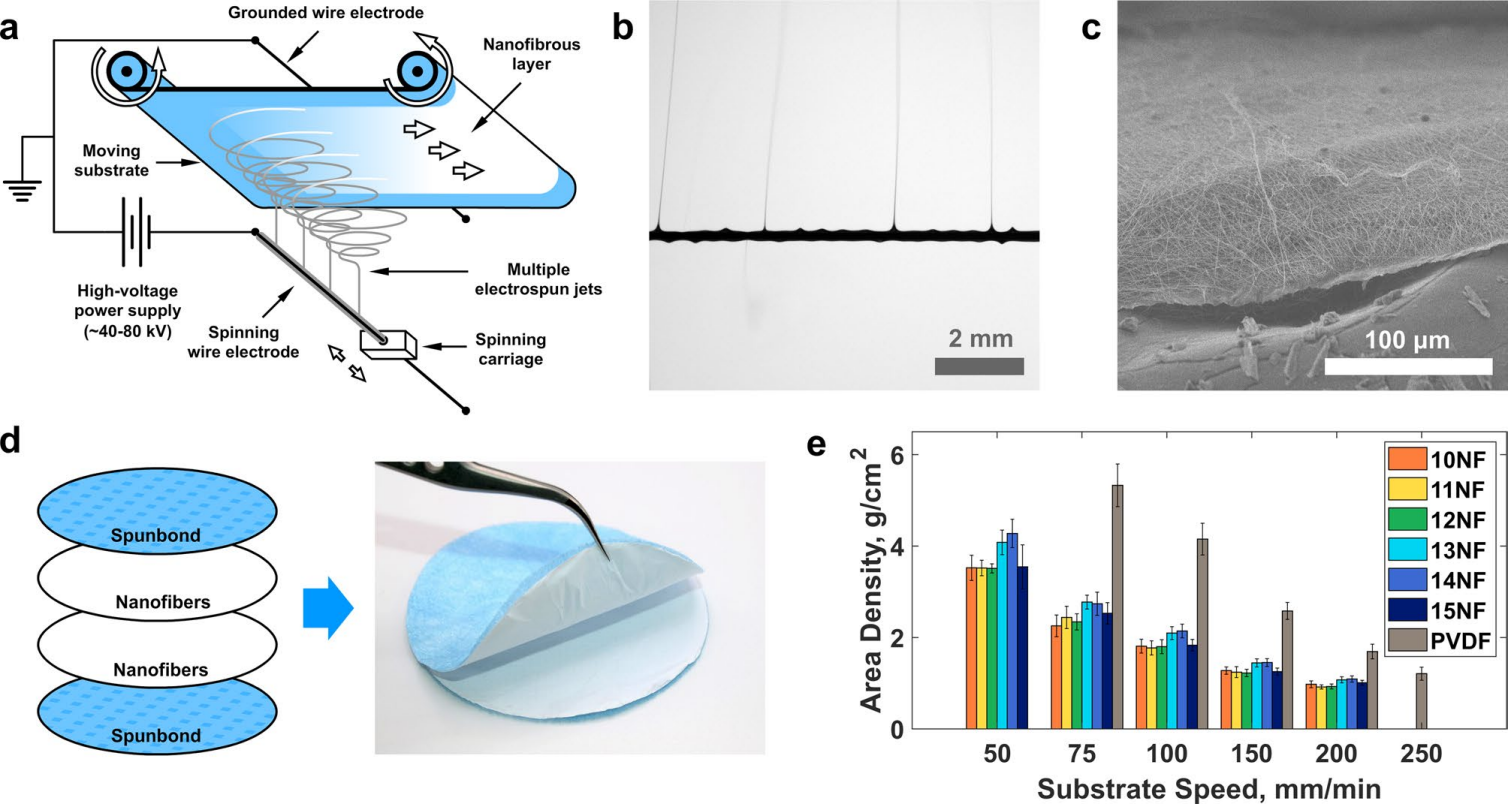
Nanofibrous filter manufacturing: (**a**) schematic representation of needleless electrospinning; (**b**) a close-up photo of electrospun jets formed uniformly along the length of the spinning wire electrode; (**c**) scanning electron micrograph of the cross-section of the resulting nanofibrous material; (**d**) schematics and photo of the assembled filter with double-folded nanofibrous layer; (**e**) mean area densities of double-folded nanofibrous layers for different substrate speeds with error bars representing the standard deviations.

Nanofibrous filters were made using the needless electrospinning technique on the Nanospider NSLAB device (Elmarco s.r.o., Liberec, Czech Republic). Six spinning solutions with concentrations from 10 to 15% w/w in 1% increments were prepared by dissolving acrylic yarn in DMF on a magnetic stirrer hotplate at 40 °C for 24 h. When each polymer solution became homogeneous, it was loaded into a spinning carriage of the Nanospider device. The carriage moved back and forth along the stationary wire spinning electrode at a constant speed, and the polymer solution flowed on the wire from a small orifice in the carriage. The voltage of 80 kV was applied to the wire electrode, and the thin film of polymer solution on its surface emitted multiple liquid jets that were accelerated and stretched by the electric field and flew towards the grounded collecting electrode in the upper part of the spinning chamber (Fig. 1a,b). Solvent quickly evaporated from the jet surface, and the resulting layer of dry fibers was collected on a spunbond nonwoven substrate placed between the electrodes at a distance of 20 mm from the collecting electrode (Fig. 1c). The diameter of the spinning electrode was 0.2 mm; the diameter of the feeding orifice in the spinning carriage was 0.6 mm; the distance between spinning and collecting electrodes was 180 mm.

The period of movement of the spinning carriage along the 35 cm wire spinning electrode was 6 s, which corresponds to a linear speed of movement of the carriage of 58.3 mm/s. This period was chosen empirically so that the formation of fibers was observed simultaneously along the entire length of the electrode, regardless of the current position of the carriage. The air parameters inside the spinning chamber are summarized in Table 1. They include relative humidity and temperature, which were measured using the Testo 635-2 thermohygrometer (Testo North America, PA, USA).

**Table 1:** Air parameters in the spinning chamber.

Group of nanofibrous filters	Polymer weight concentration, % w/w	Temperature, °C	Relative humidity, %
10NFs	10	30.9 ± 0.5	19.4 ± 0.8
11NFs	11	23.2 ± 0.1	9.3 ± 0.3
12NFs	12	29.4 ± 0.7	6.4 ± 0.4
13NFs	13	26.6 ± 1.8	9.3 ± 1.8
14NFs	14	23.0 ± 0.1	9.6 ± 0.2
15NFs	15	29.8 ± 0.7	21.4 ± 0.8
PVDF	10	32.0 ± 0.8	7.6 ± 0.5

The substrate material moved at a constant speed in the direction perpendicular to the electrodes, so the layer of nanofibers was deposited uniformly along the length of the substrate. For each polymer solution concentration, we produced five materials at substrate speeds of 50, 75, 100, 150 and 200 mm/min.

PVDF filters were made in the same manner from a spinning solution of 10% concentration in a 1:1 (w:w) mixture of DMF and acetone. This solution composition was chosen as previously used for the electrospinning of nanofiber filters from this polymer grade^36^. PVDF filters were produced at substrate speeds of 75, 100, 150, 200 and 250 mm/min, since at a lower speed of 50 mm/min, the resulting thick layer of nanofibers cracked and partially separated from the substrate under its own weight, becoming unsuitable for further testing.

After the manufacturing procedure, each material was folded in half with the substrate facing out. Thus, each nanofibrous filter consisted of a double layer of nanofibers placed between the outer layers of the spunbond to protect against mechanical damage (Fig. 1d). The resulting PAN nanofibrous filters were coded in groups by their spinning solution concentration as 10NFs, 11NFs, 12NFs, etc., and individually by their substrate speeds as 10NF-50, 10NF-75, 10NF-100, etc. Similarly, polyvinylidene fluoride nanofibrous filters have been group coded as PVDF and individually as PVDF-75, PVFD-100, etc. The filters were placed in a vacuum chamber at a pressure of 10 mmHg for 2 h to remove solvent residues and then stored for 2 days at a relative humidity of 21 ± 1.5% and temperature of 20 ± 1.2 °C.

### Characterization

#### Fiber morphology

Filters were imaged using a scanning electron microscope SNE-4500 M PLUS (NanoImages LLC, CA, USA) to measure the diameters of nanofibers, assess their alignment, and detect the electrosprayed microdroplets and bead-on-string structures. The MCM-100 sputter was used to coat the samples with gold for better image quality. Material samples were examined at 1000 × and 50,000 × magnification at six randomly selected points. The diameters of all individual fibers in the field of view were measured using the measurement tools of the microscope software. Descriptive statistics included mean, median, and geometric mean values, standard deviation, as well as shape skewness $S$, and excess kurtosis ${K}_{e}$ of a sample calculated as follows:

1 $$S=\frac{\frac{1}{n}\sum_{i=1}^{n}{\left({x}_{i}-{x}_{m}\right)}^{3}}{{\left(\frac{1}{n}\sum_{i=1}^{n}{\left({x}_{i}-{x}_{m}\right)}^{2}\right)}^\frac{3}{2}},$$

2 $${K}_{e}=\frac{\frac{1}{n}\sum_{i=1}^{n}{\left({x}_{i}-{x}_{m}\right)}^{4}}{{\left(\frac{1}{n}\sum_{i=1}^{n}{\left({x}_{i}-{x}_{m}\right)}^{2}\right)}^{2}}-3,$$

where $x_{i} ,i = \overline{{1,n}}$ are the nanofiber diameter values, $n$ is the number of measured diameters, and ${x}_{m}$ is the mean diameter.

#### Statistics

Statistical significance of the observed correlations between the solution concentration as an independent variable and the mean diameter as a dependent variable was assessed by testing the hypothesis of no correlation (i.e., the null hypothesis) against the alternative hypothesis of nonzero correlation using the one-way ANOVA test followed by Tukey’s Honest Significant Difference test with the pairwise comparison (*p*-values < 0.05 were considered statistically significant).

#### Area density of the nanofibrous layer

For each nanofibrous filter, round pieces with a diameter of 74 mm were cut at 5 points evenly distributed over the width of the nanofiber layer. The spunbond substrate material was peeled off, and the remaining double-folded layer of polymer nanofiber was weighted on the OHAUS Pioneer PX analytical balance. The resulting average weights were converted to an area of 1 m^2^ to calculate the area density (i.e., mass per unit area) of the double-folded nanofibrous layer.

#### Aerosol permeability and pressure drop

Nanofibrous filters and commercial CBRN filter were tested for aerosol penetration and pressure drop using the CertiTest Automated filter tester 3160 (TSI Inc., MN, USA). Aerosol penetration was measured as the ratio of the numbers of aerosol particles before and after passing the flat filter material counted using two condensation particle counters. The filtration efficiency was determined as

3 $$E=\left(1-p\right)\cdot 100\%.$$

We used two liquid monodisperse aerosols of dioctyl phthalate (DOP) with particle diameters of 0.185 and 0.3 µm as test aerosols. The DOP aerosol with a diameter of 0.185 µm is used for measurements of the filtration efficiency of CBRN respirator canisters^5^ and P100 particulate filters^37^. The aerosol with a 0.3 µm diameter is recommended by the NIOSH Guide to the selection and use of particulate respirators for the worst-case certification testing of the particulate filters^38^.

**Figure 2:**
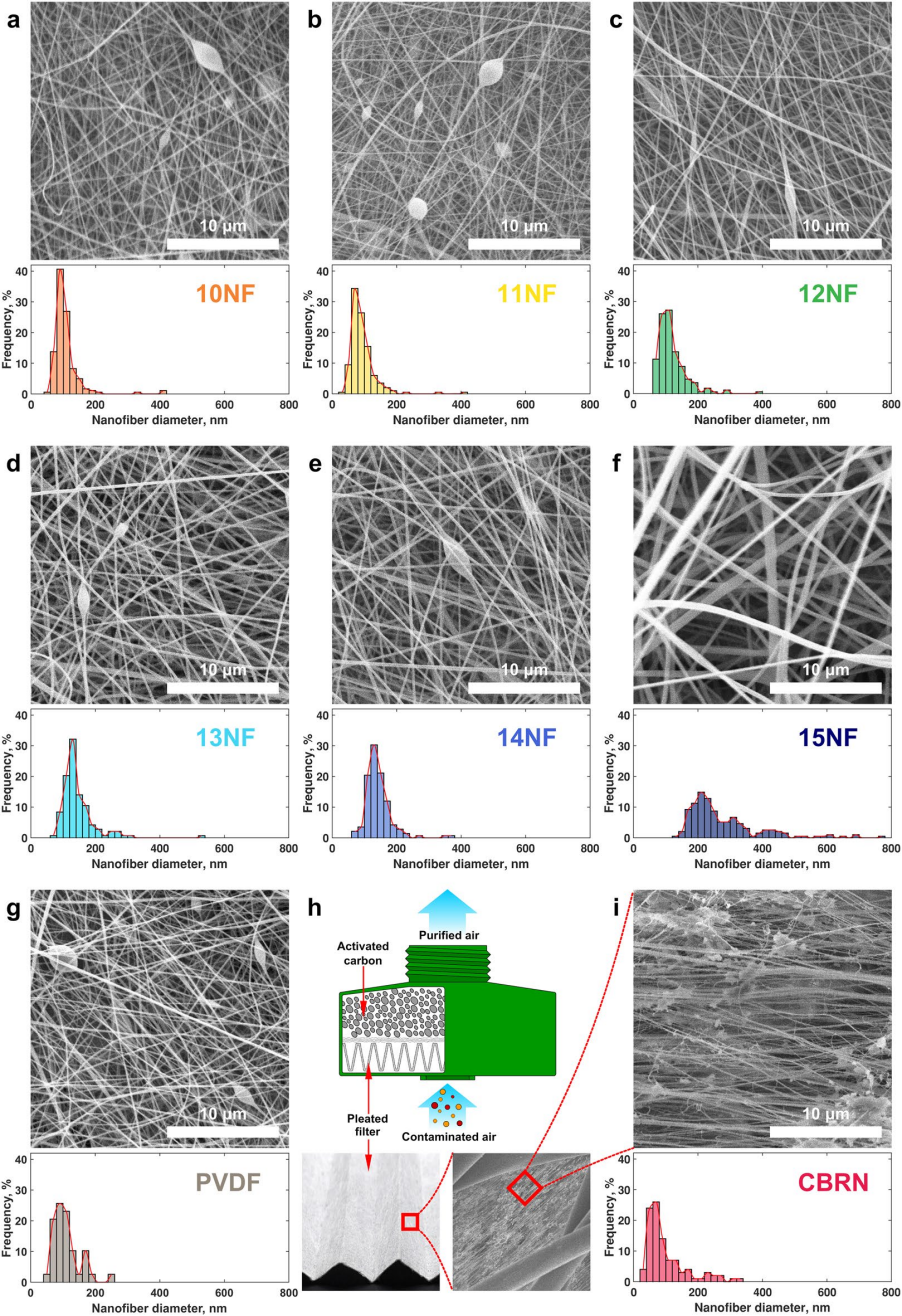
Microstructure of the aerosol filters: (**a–g,i**) scanning electron micrographs and nanofiber diameter distribution in the nanofibrous filters 10NF, 11NF, 12NF, 13NF,14NF, 15NF, PVDF, and the commercial CBRN filter, respectively; (**h**) schematics of the commercial filter canister and the photos of the pleated filter microstructure.

The Standard for CBRN Full Facepiece Air Purifying Respirator (APR) requires testing the permeability and breathing resistance of a filter at a continuous airflow rate through the canister of 85 L/min^5^. For our penetration tests, we chose the value of the face velocity of the air passing through the flat filter based on the area of the particulate filter in the CBRN filter canister. The canister consists of the sorbent bed with activated charcoal to clean the air of gaseous chemicals and the pleated particulate filter to trap aerosols (Fig. 2h). Pleats are used to increase the working area of the particulate filter and thereby reduce the breathing resistance of the device. The pleated filter was removed from the canister and stretched, and its area was measured to be approximately 735 cm^2^; however, considering that part of the filter area is covered with glue that attaches the filter to the canister wall, this value was reduced to 700 cm^2^. Thus, we determined the face velocity of the air for our penetration tests on flat filter samples as $v= \frac{85\mathrm{ l}/\mathrm{min}}{700 {\mathrm{cm}}^{2}}=2.03\,\mathrm{cm}/\mathrm{s}$. The concentrations of 0.185 and 0.3 µm aerosols were 5.5–6.2·10^5^ cm^−3^ and 2.4–2.96·10^5^ cm^−3^, respectively. Pressure drop was measured directly during the permeability test. To evaluate the integrated performance of the filters, we used the quality factor calculated as

4 $${Q}_{F}=-\frac{\mathrm{ln}\left(p\right)}{\Delta P},$$

where $p$ is the penetration, and $\Delta P$ is the pressure drop.

## Results

### Material morphology and nanofiber diameter distribution

All tested electrospun samples consisted of randomly oriented nanofibers and did not have visible defects such as holes or large drops (Fig. 2a–g). Beads were present on the fibers of all samples except 15NFs, which is consistent with the assumption that the lower concentration and viscosity of the spinning solution facilitate the formation of beads^39^. Although we observed a general trend of a decrease in the average nanofiber diameter with the reduction in the concentration of the spinning solution, the materials 10NFs had a mean diameter larger than 11NFs. Also, a sharp increase in the average diameter of the nanofibers occurred with changing concentration from 14 to 15%. The area density of the electrospun filters decreased with the increasing speed of the substrate (Fig. 1e). Electrospinning of polyvinylidene fluoride nanofibers was more productive, resulting in the higher area density of PVDF materials than PAN materials for the same substrate speeds.

We found that the commercial CBRN filter consists of outer layers of thick fibers with a mean diameter of ~ 40 μm, separated by a layer of nanofibers with a mean diameter of < 100 nm (Fig. 2h,i). Nanofibers in the CBRN filter are highly aligned and covered with adherent particles, possibly adhesive. It was not possible to measure the area density of the CBRN nanofiber layer because it was impossible to separate it from the outer layers without damage. The descriptive statistics demonstrated that nanofibers in the CBRN filter had the lowest median and geometric mean diameters, and the nanofibers in 11NFs filters had the lowest mean diameter of all filters studied (Table 2).

**Table 2:** Descriptive statistics of the nanofiber diameter distribution.

Parameters	Group of nanofibrous filters							CBRN filter
	10NFs	11NFs	12NFs	13NFs	14NFs	15NFs	Kynar	
Mean, nm	106.0	92.5	119.4	144.6	142.7	276.3	114.1	99.9
Standard deviation, nm	44.0	41.0	45.0	53.2	40.6	112.1	43.5	64.4
Median, nm	98	82.7	108	131	135	239	106	74.7
Geometric mean, nm	100.9	86.7	113.1	138.0	138.2	259.1	107.7	85.4
Skewness	4.60	3.75	2.34	3.37	2.44	1.84	2.54	1.76
Excess kurtosis	27.76	22.26	8.49	18.70	10.04	3.88	9.2	2.75

The 10NFs and 11NFs electrospun materials were closest to the commercial filter in terms of fiber diameter. Analysis of variance showed that there were no significant differences between the average diameters of nanofibers in the CBRN filter and electrospun materials 10NF, 11NF, 12NF, and PVDF (p-values > 0.21). The larger standard deviation in the CBRN filter indicates that diameters spread out over a wider range than in the electrospun filters 10NFs, 11NFs, 12NFs, and PVDF. ANOVA also showed significant differences between CBRN and 13NFs filters (p = 1.3·10^−6^), 11NFs and 12NFs (p = 1·10^−3^), and 13NFs and any of the filters 10NFs, 11NFs, 12NFs, and PVDF (p < 0.039). The diameter distributions of electrospun and CBRN filters were positively skewed, and the CBRN filter had the lowest skewness and excess kurtosis.

### Filtration performance

**Figure 3:**
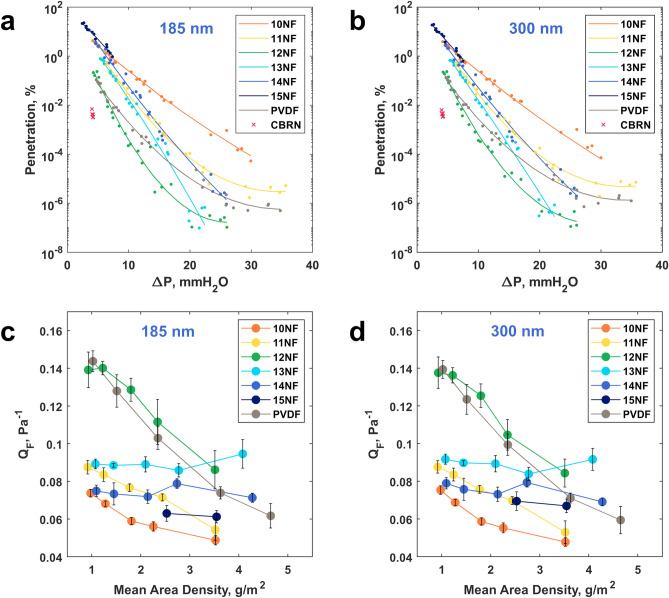
Filtration performance of the electrospun nanofibrous filters: (**a,b**) scatter plots for measured aerosol penetration and corresponding pressure drops of filter material samples fitted with second-order polynomial curves; (**c,d**) quality factors of nanofibrous filters with various average area densities corresponding to different substrate speeds with error bars representing the standard deviations.

The results of aerosol permeability tests for all groups of nanofibrous filters are summarized in Fig. 3. Materials 15NFs were only tested for the two lowest substrate speeds of 50 and 75 mm/min and had very low pressure drops and filtration efficiency, so thinner samples obtained at higher substrate speeds were not considered due to their apparent inefficiency. Materials 12NF-50 and 13NF-50 demonstrated the highest ability to retain submicron aerosols. For 12NF-50, the mean penetrations were 2.01·10^−7^% for 0.185 µm and 3.05·10^−7^% for 0.3 µm aerosols with the corresponding pressure drops < 26 mm H_2_O. For 13NF-50, the mean penetrations were 4.2·10^−7^% for 0.185 µm and 6.9·10^−7^% for 0.3 µm aerosols, and the pressure drop was < 22.5 mm H_2_O. Despite similar performance at the highest area densities and efficiencies, 12NFs materials had significantly lower breathing resistance than 13NFs materials in the penetration range of 0.000001–0.01%. Material PVDF-75 had mean penetrations of 6.8·10^−7^ and 1.3·10^−6^% for 0.185 µm and for 0.3 µm aerosols, respectively, with higher breathing resistance of < 31.6 mm H_2_O. However, the breathing resistance and efficiencies of PVDF and 12NFs materials are almost equal at pressure drops below 10 mmH_2_O.

For the CBRN filter material, a five-fold spread in permeability was observed with an average penetration of 4.4·10^−3^% for both aerosols, while the pressure drop was stable in the range of 4.0–4.2 mm H_2_O. Comparable filtration efficiency of the 12NFs materials was achieved at a higher pressure drop of 7.0–7.5 mm H_2_O. For all nanofibrous materials and CBRN filter, the filtration efficiencies for 0.185 and 0.3 µm aerosols differed by less than an order of magnitude.

Fitting curves demonstrated a linear relationship on a logarithmic scale between pressure drop and filtration efficiency for materials 10NFs, 13NFs, and 14NFs (Fig. 3a,b). For materials 11NFs and 12NFs, this relationship was non-linear, and the pressure drop increased without improving efficiency at higher area densities. The mutual position of the fitted curves indicated that for the same penetration values, the breathing resistance decreases with an increase in the concentration of the spinning solution from 10 to 12%. The opposite effect of increasing pressure drop was observed with a further increase in the concentration above 12%.

The materials of the 12NFs group had the best quality factor among all PAN nanofiber filters, except for the sample 12NF-50 with the highest area density, which was slightly inferior to the sample 13NF-50 (Fig. 3c,d). The ${Q}_{F}$ reached its maximum of ~ 0.14 Pa^−1^ at the low area densities of 12NFs, decreasing with layer thickening. A similar effect was observed for materials 10NFs and 11NFs, while ${Q}_{F}$ was relatively constant for 13NFs and 14NFs. PVDF filters exhibit similar quality factors as 12NFs materials, slightly inferior to the latter at higher area densities, but exceeding at the lowest densities. The CBRN filter had significantly higher quality factors of 0.249 ± 0.0048 Pa^−1^ and 0.247 ± 0.0047 Pa^−1^ for 0.185 μm and 0.3 μm aerosols, respectively.

## Discussion

Our study demonstrates that ultra-fine electrospun fibers can trap submicron particles with an efficiency of > 99.999999%, thereby reducing penetration by more than 30,000-fold compared to the minimum allowable efficiency of 99.97% for CBRN respirators^5,37^. The corresponding breathing resistance of this filter was 2.5 times lower than the NIOSH-approved limit of 65 mmH_2_O for CBRN respirators with face-mounted canisters. These results demonstrate that highly-effective mechanical retention of 0.185 and 0.3 µm aerosols by nanofibrous media can be achieved at an average fiber diameter < 150 nm. In contrast, 15NFs nanofibrous materials with a mean diameter of 276 nm and larger inter-fiber spacing had the lowest airflow resistance but could not effectively capture aerosols. The proportional dependence of the diameter of the electrospun nanofibers on the concentration of a spinning solution is well known^40^. Nevertheless, our 10NFs materials had a larger mean fiber diameter than the 11NFs, which can be explained by the increased air humidity inside the chamber during electrospinning (Table 1). The critical parameters that determine the final diameter of the fibers include not only the concentration of the polymer, which determines the rheological properties in the spinning solution, but also the rate of evaporation of the volatile solvent, which makes up the bulk of the initial polymer jet. Higher humidity slows down the evaporative mass transfer through the jet’s surface, leading to a slower jet diameter reduction during its flight toward the substrate and, ultimately, to the formation of thicker nanofibers. The same can explain a sharp jump in the average fiber diameter of 15NFs materials compared to the 14NFs.

Despite the high filtration efficiency, our nanofibrous filters were inferior to the commercial filter in terms of their quality factor. The quality factor illustrates the overall performance of a filter by combining how effectively the filter traps aerosol and how easy it is to breathe through. Previous studies demonstrated the role of complex morphology on the quality factor of electrospun materials^41,42^. The descriptive statistics and results of penetration tests reveal that simply reducing the average diameter is not enough to achieve high performance in a nanofibrous filter, and the shape of fiber diameter distribution has a decisive influence on filtration efficiency.

Although there is no statistically significant difference between the average fiber diameter in the CBRN filter and 10NFs, 11NFs, 12NFs, and PVDF electrospun filters, the quality factor of the CBRN filter is still higher. The smallest skewness of the fiber diameter distribution of the CBRN filter indicates that it is closest to the normal distribution (S = 0), and its smallest kurtosis suggests that the diameter distribution produces fewer outliers. Likewise, the best quality factor among electrospun materials was observed in 12NFs, which had lower values of skewness and excess kurtosis of fiber diameter distribution than the other electrospun materials with mean diameters < 150 nm. A positive skewness and high kurtosis in nanofibrous filters indicate that the right tail of its diameter distribution is much larger than the left. Thus, a significant number of fibers were thicker than the average. Local thickening of the fibers leads to an increase in the pore size resulting in a lower probability of aerosol retention in the corresponding spots of the filter, degrading overall filtration efficiency. This local degradation is critical for ultra-low penetration filters when even a relatively small number of particles passing through the filter worsens its efficiency by orders of magnitude. It should be noted that the CBRN filter has a standard deviation of fiber diameters greater than that of electrospun filters, and this wider spread of values around the peak does not degrade filtration performance. It is noteworthy that PVDF and 12NFs filters perform approximately the same, while their skewness and kurtosis are close to each other.

All of the above allows speculation that electrospun filters with a higher quality factor should have closer to normal diameter distribution and fewer oversized fibers, but this statement should be verified by repeating the same tests on nanofibrous materials with various fiber diameter distributions, including sizeable positive and negative skewnesses and different amounts of outliers. In addition, the role of nanofiber alignment in the filtration process also needs to be clarified. Filtration performance is generally considered independent of the in-plane orientation of the micro-scale fibers^43,44^. On the contrary, some studies claim that the alignment of nanofibers in filtration nonwovens can improve their performance by increasing aerosol capture efficiency and reducing pressure drop^45^. The effect of the beaded structure of nanofibrous media on filtration also needs to be studied, as improvements in filter quality factor due to the presence of beads have been previously reported^29^. Further, since the electrostatic mechanism contributes significantly to the retention of aerosols, it must be considered when comparing filter efficiency. Finally, we should not disregard the chemical and physical properties of the various fiber-forming polymers, which can affect airborne particle capture through intermolecular forces or chemical bonding.

In summary, the electrospun nanofibers have shown a high potential for use in high-efficient respiratory protective equipment, and the presented results can inform future studies of nanofibrous filters’ effectiveness in retaining submicron aerosols. Better control over the nanofiber diameter distribution can improve their protective and ergonomic properties.

## Data Availability

The datasets used or analyzed during the current study are available from the corresponding author on reasonable request.

## References

[1] Richardt A, Hülseweh B, Niemeyer B, Sabath F (2013). CBRN Protection: Managing the Threat of Chemical, Biological, Radioactive and Nuclear Weapons.

[2] Podgórski A, Bałazy A, Gradoń L (2006). Application of nanofibers to improve the filtration efficiency of the most penetrating aerosol particles in fibrous filters. Chem. Eng. Sci..

[3] Serfozo N, Ondráček J, Zíková N, Lazaridis M, Ždímal V (2017). Size-resolved penetration of filtering materials from CE-marked filtering facepiece respirators. Aerosol. Air Qual. Res..

[4] Brown RC (1993). Air Filtration: An Integrated Approach to the Theory and Applications of Fibrous Filters.

[5] National Institute for Occupational Safety and Health. *Statement of Standard for Chemical, Biological, Radiological, and Nuclear (CBRN) Full Facepiece Air Purifying Respirator (APR)*. (2003).

[6] *Electrospun nanofibers for energy and environmental applications.* (Springer, 2014).

[7] Reneker DH, Yarin AL (2008). Electrospinning jets and polymer nanofibers. Polymer (Guildf).

[8] *Advanced Nanofibrous Materials Manufacture Technology Based on Electrospinning*. (Taylor & Francis Group, an Informa Business, 2019).

[9] Yu M (2017). Recent advances in needleless electrospinning of ultrathin fibers: From academia to industrial production. Macromol. Mater. Eng..

[10] Wang L, Zhang C, Gao F, Pan G (2016). Needleless electrospinning for scaled-up production of ultrafine chitosan hybrid nanofibers used for air filtration. RSC Adv.

[11] Chen H, Huang M, Liu Y, Meng L, Ma M (2020). Functionalized electrospun nanofiber membranes for water treatment: A review. Sci. Total Environ..

[12] Karabulut FNH, Höfler G, Chand NA, Beckermann GW (2021). Electrospun nanofibre filtration media to protect against biological or nonbiological airborne particles. Polymers (Basel).

[13] Blosi M (2021). Polyvinyl alcohol/silver electrospun nanofibers: Biocidal filter media capturing virus-size particles. J. Appl. Polym. Sci..

[14] Augustine R (2020). Electrospun chitosan membranes containing bioactive and therapeutic agents for enhanced wound healing. Int. J. Biol. Macromol..

[15] Zhu M (2017). Electrospun nanofibers membranes for effective air filtration. Macromol. Mater. Eng..

[16] Li Y, Yin X, Yu J, Ding B (2019). Electrospun nanofibers for high-performance air filtration. Compos. Commun..

[17] Mamun A, Blachowicz T, Sabantina L (2021). Electrospun nanofiber mats for filtering applications—Technology. Struct. Mater. Polymers (Basel).

[18] Zhang Z, Ji D, He H, Ramakrishna S (2021). Electrospun ultrafine fibers for advanced face masks. Mater. Sci. Eng. R. Rep..

[19] Lu T (2021). Multistructured electrospun nanofibers for air filtration: A review. ACS Appl. Mater. Interfaces.

[20] Lyu C (2021). Electrospinning of nanofibrous membrane and its applications in air filtration: A review. Nanomaterials (Basel).

[21] Essa WK, Yasin SA, Saeed IA, Ali GAM (2021). Nanofiber-based face masks and respirators as covid-19 protection: A review. Membranes (Basel).

[22] Wang H (2021). Development of electrospun nanofibrous filters for controlling coronavirus aerosols. Environ. Sci. Technol. Lett..

[23] Yang S (2007). Aerosol penetration properties of an electret filter with submicron aerosols with various operating factors. J. Environ. Sci. Health A Tox Hazard Subst Environ. Eng..

[24] Xiao H, Chen G, Song Y (2012). Penetration performance of melt-blown polypropylene electret nonwoven web against DEHS aerosols. Adv. Mater. Res..

[25] Tsai P (2020). Performance of masks and discussion of the inactivation of SARS-CoV-2. Eng. Sci..

[26] Hossain E (2020). Recharging and rejuvenation of decontaminated N95 masks. Phys. Fluids.

[27] Ullah S (2020). Reusability comparison of melt-blown vs. nanofiber face mask filters for use in the coronavirus pandemic. ACS Appl. Nano Mater..

[28] Gudgin Dickson EF (2013). Personal Protective Equipment for Chemical, Biological, and Radiological Hazards: Design, Evaluation, and Selection.

[29] Wang Z, Zhao C, Pan Z (2015). Porous bead-on-string poly(lactic acid) fibrous membranes for air filtration. J. Colloid Interface Sci..

[30] Wang T, Kumar S (2006). Electrospinning of polyacrylonitrile nanofibers. J. Appl. Polym. Sci..

[31] Isaac B, Taylor RM, Reifsnider K (2020). Anisotropic characterizations of electrospun PAN nanofiber mats using design of experiments. Nanomaterials.

[32] Huang ZM, Zhang YZ, Kotaki M, Ramakrishna S (2003). A review on polymer nanofibers by electrospinning and their applications in nanocomposites. Compos. Sci Technol..

[33] Teo WE, Ramakrishna S (2006). A review on electrospinning design and nanofibre assemblies. Nanotechnology.

[34] Joint Service General Purpose Mask (JSGPM) M-50/M-51 - USAASC. https://asc.army.mil/web/portfolio-item/jpeo-cbd-joint-service-general-purpose-mask-jsgpm-m-50m-51/. Accessed 1 December 2022.

[35] Lukanina KI (2021). Efficiency of respiratory protective equipment in the SARS-CoV-2 pandemic. Nanobiotechnol. Rep..

[36] Lolla D, Pan L, Gade H, Chase GG, Tański T, Jarka P, Matysiak W (2018). Functionalized polyvinylidene fluoride electrospun nanofibers and applications. Electrospinning Method Used to Create Functional Nanocomposites Films.

[37] National Institute for Occupational Safety and Health. 42 CFR § 84.174 - Filter efficiency level determination test - non-powered series N, R, and P filtration. https://www.ecfr.gov/current/title-42/chapter-I/subchapter-G/part-84/subpart-K/section-84.174. Accessed 1 December 2022.

[38] National Institute for Occupational Safety and Health. NIOSH Guide to the Selection and Use of Particulate Respirators. *DHHS (NIOSH) Publication Number 96–101(1996)* https://www.cdc.gov/niosh/docs/96-101/default.html. Accessed 1 December 2022.

[39] Fong H, Chun I, Reneker DH (1999). Beaded nanofibers formed during electrospinning. Polymer (Guildf).

[40] Chen X (2019). Electrospinning on a plucked string. J. Mater. Sci..

[41] Cao Q (2019). Electrospun bead-in-string fibrous membrane prepared from polysilsesquioxane-immobilising poly(lactic acid) with low filtration resistance for air filtration. J. Polym. Res..

[42] Zhou M (2019). Large-scale preparation of micro-gradient structured sub-micro fibrous membranes with narrow diameter distributions for high-efficiency air purification. Environ. Sci. Nano.

[43] Tahir MA, Vahedi Tafreshi H (2009). Influence of fiber orientation on the transverse permeability of fibrous media. Phys. Fluids.

[44] Fotovati S, Vahedi Tafreshi H, Pourdeyhimi B (2010). Influence of fiber orientation distribution on performance of aerosol filtration media. Chem. Eng. Sci..

[45] Akampumuza O (2019). Analyzing the effect of nanofiber orientation on membrane filtration properties with the progressive increase in its thickness: A numerical and experimental approach. Text. Res. J..

